# High-field MR imaging in pediatric congenital heart disease: Initial results

**DOI:** 10.1007/s00247-014-3093-y

**Published:** 2014-08-03

**Authors:** Kim-Lien Nguyen, Sarah N. Khan, John M. Moriarty, Kiyarash Mohajer, Pierangelo Renella, Gary Satou, Ihab Ayad, Swati Patel, M. Ines Boechat, J. Paul Finn

**Affiliations:** 1 Division of Cardiology, VA Greater Los Angeles Healthcare System, David Geffen School of Medicine at UCLA, Los Angeles, CA USA; 2 Department of Radiological Sciences, University of California at Los Angeles, Peter V. Ueberroth Bldg., Ste. 3371, 10945 Le Conte Ave., Los Angeles, CA 90095-7206 USA; 3 Division of Pediatric Cardiology, David Geffen School of Medicine at UCLA, Los Angeles, CA USA; 4 Department of Anesthesia, David Geffen School of Medicine at UCLA, Los Angeles, CA USA

**Keywords:** Magnetic resonance imaging, Magnetic resonance angiography, 3.0 Tesla, Congenital heart disease, Cardiovascular, Neonate, Children

## Abstract

**Background:**

Comprehensive assessment of pediatric congenital heart disease (CHD) at any field strength mandates evaluation of both vascular and dynamic cardiac anatomy for which diagnostic quality contrast-enhanced magnetic resonance angiography (CEMRA) and cardiac cine are crucial.

**Objective:**

To determine whether high-resolution (HR) CEMRA and steady-state free precession (SSFP) cine can be performed reliably at 3.0 T in children with CHD and to compare the image quality to similar techniques performed at 1.5 T.

**Materials and methods:**

Twenty-eight patients with a median age of 5 months and average weight 9.0 ± 7.8 kg with suspected or known CHD were evaluated at 3.0 T. SSFP cine (*n* = 86 series) and HR-CEMRA (*n* = 414 named vascular segments) were performed and images were scored for image quality and artifacts. The findings were compared to those of 28 patients with CHD of similar weight who were evaluated at 1.5 T.

**Results:**

Overall image quality on HR-CEMRA was rated as excellent or good in 96% (397/414) of vascular segments at 3.0 T (k = 0.49) and in 94% (349/371) of vascular segments at 1.5 T (k = 0.36). Overall image quality of SSFP was rated excellent or good in 91% (78/86) of cine series at 3.0 T (k = 0.55) and in 81% (87/108) at 1.5 T (k = 0.47). Off-resonance artifact was common at both field strengths, varied over the cardiac cycle and was more prevalent at 3.0 T. At 3.0 T, off-resonance dark band artifact on SSFP cine was absent in 3% (3/86), mild in 69% (59/86), moderate in 27% (23/86) and severe in 1% (1/86) of images; at 1.5 T, dark band artifact was absent in 16% (17/108), mild in 69% (75/108), moderate in 12% (13/108) and severe in 3% (3/108) of cine images. The signal-to-noise ratio and contrast-to-noise ratio of both SSFP cine and HR-CEMRA images were significantly higher at 3.0 T than at 1.5 T (*P* < 0.001).

**Conclusion:**

Signal-to-noise ratio and contrast-to-noise ratio of high-resolution contrast-enhanced magnetic resonance angiography and SSFP cine were higher at 3.0 T than at 1.5 T. Artifacts on SSFP cine were cardiac phase specific and more prevalent at 3.0 T such that frequency-tuning was required in one-third of exams. In neonates, high spatial resolution CEMRA was highly reliable in defining extracardiac vascular anatomy.

## Introduction

Cardiac magnetic resonance imaging (MRI) and contrast-enhanced MR angiography (CEMRA) have become mainstream clinical tools during the past decade with applications in pediatric and adult congenital heart disease (CHD) [1, 2]. Whereas the vast majority of reports on cardiac MRI/MRA have focused on 1.5 T, market data as far back as 2004 indicate that 3.0-T systems make up 25% of new MRI purchases in the United States [3]. Since its approval for whole-body imaging by the United States Food and Drug Administration, 3.0-T systems have become the standard for neurological and musculoskeletal imaging at many institutions [4]. Despite high signal-to-noise ratio and increasing availability, adoption of cardiac imaging at 3.0 T has been slow, largely due to the more troublesome off-resonance artifacts on steady-state free precession (SSFP) cine images [5, 6]. Whereas in adults, several studies have addressed the advantages of 3.0 T for high spatial CEMRA [5–7], to the best of our knowledge, there are no published reports specifically addressing imaging of pediatric CHD at 3.0 T compared to 1.5 T.

The purpose of our study, therefore, was to determine whether both high-resolution (HR) CEMRA and diagnostic-quality SSFP cardiac cine can be acquired reliably in pediatric CHD patients at 3.0 T, and to compare objective and subjective measures of image quality to similar techniques at 1.5 T.

## Materials and methods

### Patient population

Twenty-eight patients (17 females, median age of 5 months [range: 3 days to 8 years] and weight 9.0 ± 7.8 kg [range: 1.4–25.0 kg]) with a range of known or suspected congenital cardiovascular disorders (Table 1) were evaluated at 3.0 T. To serve as a control group, the MRI/MRA studies of 28 patients (18 females, median age 30 months [range: 2 days to 7 years] and weight 9.6 ± 6.4 kg) with a similar spectrum of CHD disorders imaged at 1.5 T in a similar time frame were also evaluated. Of the 28 subjects, two were imaged at both 3.0 T and 1.5 T. In one subject, the interval between scans was one year and in the other the interval was three years. Because higher-resolution acquisition matrices are better tolerated for CEMRA at 3.0 T than at 1.5 T [8], the smallest patients requiring the most detailed extracardiac vascular imaging were preferentially imaged at 3.0 T. Where detailed evaluation of intracardiac anatomy was of primary concern, imaging was preferentially performed at 1.5 T. Of 28 patients imaged at 3.0 T, 23 patients had SSFP cine imaging and 28 patients had HR-CEMRA imaging. Similarly, of 28 patients imaged at 1.5 T, 26 patients had SSFP cine imaging and 25 patients had HR-CEMRA at 1.5 T. Primary study indications were: 1) assessment of thoracic vasculature (3 T: *n* = 20; 1.5 T: *n* = 20), 2) intracardiac anatomy and function (3.0 T: *n* = 17; 1.5 T: *n* = 20), 3) postsurgical sequelae (3 T: *n* = 10; 1.5 T: *n* = 11), and 4) pre-interventional or surgical planning (3.0 T: *n* = 5; 1.5 T: *n* = 4). Our study was approved by the Institutional Review Board and was compliant with the Health Insurance Portability and Accountability Act.

**Table 1:** Spectrum of pathology

1.5 T (*n* = 28)	3.0 T (*n* = 28)
Anomalous pulmonary venous return (*n* = 4)	Anomalous pulmonary venous return (*n* = 2)
Aortic coarctation (*n* = 1)	Aortic coarctation (*n* = 2)
ASD, interrupted arch, VSD, s/p arch repair & Kono/Ross procedure (*n* = 1)	AV canal defect, hypoplastic aortic arch (*n* = 1)
	Bicuspid aortic valve (*n* = 1)
Congenital valvar/supravalvar aortic stenosis s/p Ross procedure with RV to PA conduit (*n* = 1)	Crisscross heart s/p PA band and Glenn shunt (*n* = 1)
	Double outlet right ventricle (*n* = 4)
Double outlet right ventricle (*n* = 4)	Hypoplastic preductal aortic arch (*n* = 1)
Endocardial cushion defect (*n* = 1)	
Familial cardiomyopathy (*n* = 1)	Interrupted aortic arch, VSD (*n* = 1)
Heterotaxy with left atrial isomerism (*n* = 1)	Interventricular mass (*n* = 1)
	Major aortopulmonary collateral artery (*n* = 1)
Hypoplastic left heart syndrome (*n* = 3)	
Marfan with dilated root & MVP (*n* = 1)	Pulmonary atresia (*n* = 2)
	Pulmonary AVM (*n* = 1)
s/p ASD closure, muscular VSD (*n* = 1)	Right aortic arch with vascular ring (*n* = 2)
Tetralogy of Fallot (*n* = 4)	
Tricuspid atresia; s/p Stansel procedure & Glenn shunt (*n* = 1)	s/p Aortic coarctation repair (*n* = 1)
	Tetralogy of Fallot (*n* = 5)
Ventricular cardiac mass (*n* = 1)	Unbalanced AV canal defect, heterotaxy, hypoplastic arch, s/p modified Norwood & Kawashima procedure (*n* = 1)
VSD, interrupted arch s/p Norwood & Rastelli (*n* = 2)	
Widened patent ductus arteriosus (*n* = 1)	
	Ventricular cardiac mass (*n* = 1)

*ASD* atrial septal defect, *AV* atrioventricular, *AVM* arteriovenous malformation, *LV* left ventricle, *MVP* mitral valve prolapse, *s*/*p* status post, *PA* pulmonary artery, *PV* pulmonary vein, *RV* right ventricle, *VSD* ventricular septal defect

### MRI acquisition

MRI was routinely performed with assisted ventilation and controlled apnea, as previously described [9]. Neonatal (*n* = 7 at 3.0 T; *n* = 1 at 1.5 T) or pediatric (*n* = 4 at 3.0 T; *n* = 3 at 1.5 T) intensive care staff transported patients who were already intubated and monitored them during the exam. Otherwise, a dedicated pediatric anesthesiologist provided anesthesia and patient monitoring in the MRI suite (*n* = 17 at 3.0 T; *n* = 24 at 1.5 T). An MRI–compatible monitoring system (Magnitude 3159 MRI patient monitor; InVivo Research, Orlando, FL) was employed throughout the procedure for physiological monitoring (electrocardiography, pulse oximetry, and noninvasive measurement of blood pressure and end-tidal CO_2_ level) [10]. Average heart rate during image acquisition was 110 ± 27 bpm (range: 74–160 bpm) at 3.0 T and 100 ± 26 bpm (range: 47–140 bpm) (*P* = 0.26) at 1.5 T.

At 3.0 T, MR imaging was performed on a 32-channel system (Magnetom TIM Trio; Siemens Medical Solutions, Malvern, PA) with maximum gradient amplitude 40 mT/m and slew rate 200 mT/m/ms. At 1.5 T, MR imaging was performed on a 32-channel system (Magnetom TIM Avanto; Siemens Medical Solutions, Malvern, PA) with gradient amplitude = 45 mT/m and slew rate = 200 mT/m/ms. Throughout the study period, both MR systems had similar software levels and radiofrequency infrastructure. Receiver coil combinations were chosen based on body size and included head or extremity coils for neonates; flex and spine coils for infants, and body array and spine coils for larger children. Specific imaging parameters are outlined in Table 2. Routine protocols included breath-held SSFP cine MRI (Table 2) [11] and HR-CEMRA, supplemented in specific cases by targeted myocardial tagging [12], and/or phase contrast velocity measurement [13]. Depending on the indication for the study, some aspects of the examination were expanded or truncated on an individual patient basis.

**Table 2:** Technical parameters

	1.5 T		3.0 T	
	*SSFP cine*	*HR*-*MRA*	*SSFP cine*	*HR*-*MRA*
TR per line (ms)	3.1 – 3.8	2.7 – 3.3	3.0 – 3.8	2.8 – 3.5
TE (ms)	1.3 – 1.8	1.0 – 1.3	1.3 – 1.6	1.0 – 1.4
Flip angle (°)	60 – 80	12 – 30	38 – 50	14 – 20
Bandwidth (Hz/pixel)	888 – 1130	610 – 698	925 – 930	579 – 635
Field of view (mm^2^)	113 – 225	121 – 500	90 – 244	131 – 312
	x	x	x	x
	199 – 371	196 – 500	180 – 340	280 – 500
Acquisition matrix	85 – 150	448 – 576	96 – 162	210 – 328
	x	x	x	x
	192 – 256	140 – 346	192 – 208	512 – 640
Slice thickness (mm)	3.5 – 6.0	0.8 – 1.3	3.0 – 6.0	0.7 – 0.9
In-plane resolution (mm^2^)	1.0 – 1.7	0.8 – 1.3	0.9 – 1.6	0.6 – 1.0
	x	x	x	x
	0.8 – 1.0	0.7 – 1.0	0.9 – 1.8	0.5 – 1.0
Parallel imaging factor	2	2 – 4	2	2 – 4
Number of signal averages	1	1	1	1
Specific absorption rate (W/kg)	2.5 ± 0.9	2.1 ± 0.7	2.8 ± 1.3	2.5 ± 1.3
Acquisition time	7.5 ± 2.0 (sec per slice)	20.6 ± 3.7^†^	5.8 ± 1.7 (sec per slice)	21.2 ± 3.2^†^

*Values are reported as minimum – maximum. Where relevant, values are reported as mean ± SD. For 1.5 T: Magnetom TIM Avanto (Siemens Medical Solutions; Malvern, PA); gradient strength = 45 mT/m, slew rate = 200 mT/m/ms. For 3.0 T: Magnetom TIM Trio (Siemens Medical Solutions; Malvern, PA), gradient strength = 40 mT/m, slew rate = 200 mT/m/ms

†Scan time for CE-MRA sequence reflects seconds/acquisition

‡ *sec* seconds, *TE* echo time, *TR* repetition time

At 3.0 T, an initial horizontal long axis SSFP cine was performed to assess the severity of off-resonance dark band artifact at baseline [14]. If artifact was present, a patient-specific frequency offset was employed, based on a breath-held frequency scout sequence [15]. Thereafter, routine short axis and long axis cine images were acquired using the optimized frequency offset. If dark band artifact recurred in other orientations, the frequency offset was readjusted based on the updated image plane.

The HR-CEMRA acquisition was planned on the basis of a timing run performed with a time-resolved 3-D TWIST (Time Resolved Imaging with Stochastic Trajectories [16]) sequence. TWIST images were obtained in the coronal orientation simultaneously with administration of one-sixth of the total dose of gadopentetate dimeglumine (Magnevist; Bayer, Leverkusen, Germany; 3.0 T: *n* = 16; 1.5 T: *n* = 20) or gadobenate dimeglumine (MultiHance; Bracco Diagnostics, Princeton, NJ; 3.0 T: *n* = 11; 1.5 T: *n* = 7) or gadofoveset trisodium (Ablavar; Lantheus Medical Imaging, N. Billerica, MA; 3.0 T: *n* = 1; 1.5 T: *n* = 1). The TWIST images were used mainly for timing purposes and were excluded from our image quality analysis. HR-CEMRA was performed with a spoiled 3-D gradient echo sequence with parameters as detailed in Table 2.

The respective contrast agents were diluted with saline by a factor of 3–7 and the solution was infused at a rate of 0.2-0.8 mL/s (depending on patient size) using an electronic injector (Spectris Solaris; Medrad, Warrendale, PA). The dilution factor was calculated based on delivery of the contrast bolus over a 15-s infusion window (approximately 75% of the data acquisition window) at the specified, patient-specific infusion rate. Total gadolinium dosage was prescribed at 0.2 mmol/kg for extracellular contrast agents (Magnevist and MultiHance) and 0.06 mmol/kg for gadofoveset (Ablavar), corresponding to double the FDA-approved single dose for each respective agent. The same contrast dilution factor and infusion rate was employed for TWIST and HR-CEMRA in a given patient.

### Image analysis

Three board-certified radiologists and one pediatric cardiologist with training in cardiovascular imaging independently scored the images. All studies were randomized and all readers were blinded to the field strength used for imaging, clinical indication for the examination, as well as correlative data during the scoring process.

#### Qualitative Analysis of SSFP Cine Images

Image quality of long axis (LAX) views and representative basal, mid-ventricular, and apical slices from the short axis (SAX) view were scored on a four-point scale (1 = poor--nondiagnostic with poorly defined borders and ill-defined intracardiac detail; 2 = fair--diagnostic with some degradation and blurring of cardiac borders; 3 = good--diagnostic with defined borders and good contrast, and 4 = excellent--diagnostic with well-defined borders and high myocardium to blood pool contrast). Image artifacts were also scored on a four-point scale (0 = no artifacts; 1 = mild, artifacts not interfering with diagnostic content; 2 = moderate, artifacts degrading diagnostic content, and 3 = severe, artifacts resulting in nondiagnostic images). Types of artifacts were identified as resulting from pulsatile flow, off-resonance/banding and metal artifacts. Twenty-three patients had cine imaging at 3.0 T and 26 patients had cine imaging at 1.5 T. A total of 86 SSFP image series (34 LAX and 52 SAX) acquired at 3.0 T and 108 SSFP image series (38 LAX and 70 SAX) acquired at 1.5 T were analyzed.

#### Qualitative Analysis of HR-CEMRA

Vessel border definition was scored per segment using a four-point scale (1 = poor—cannot be confidently evaluated; 2 = fair—can be evaluated for structural pathology with moderate confidence; 3 = good—can be evaluated for structural pathology with high confidence, and 4 = excellent—sharp vessel borders and fine detail can be confidently evaluated). Thoracoabdominal vessels were divided into 15 segments: ascending aorta, transverse aorta, descending aorta, subclavian artery, carotid artery, pulmonary trunk, main pulmonary artery, pulmonary arterial branches, main pulmonary veins, pulmonary venous tributaries, abdominal aorta, celiac trunk, superior mesenteric artery, renal arteries and iliac arteries. Of 420 vascular segments (*n* = 28 patients) imaged at 3.0 T, 414 vascular segments were available for analysis (*n* = 6 segments were excluded [*n* = 2 absent pulmonary artery trunk, *n* = 1 absent main pulmonary artery, *n* = 1 absent pulmonary arterial branches, *n* = 2 iliac arteries with insufficient coil coverage]). Of 375 segments (*n* = 25 patients; 3 patients did not have HR-CEMRA) imaged at 1.5 T, 371 segments were analyzed (*n* = 4 were segments were excluded [*n* = 1 absent main pulmonary artery, *n* = 1 absent pulmonary artery trunk, *n* = 1 immature renal arteries, *n* = 1 iliac arteries with insufficient coil coverage]). Artifacts were classified as resulting from physiological motion, metal wires or implants, suboptimal bolus timing or parallel acquisition reconstruction. Overall artifact per image was scored on a four-point scale as described in the section on qualitative analysis of SSFP cine images.

#### Quantitative analysis

Signal-to-noise ratio and contrast-to-noise ratio of the left ventricular myocardium were determined using the mid-ventricular SAX slice at the level of the papillary muscle at end-diastole. A region of interest (ROI) of at least 0.2 cm was measured outside the patient, with the standard deviation defined as noise. Because noise is non-uniformly distributed in parallel imaging [17–19], an average of three ROIs was used. The myocardial signal intensity was defined as the mean signal from a semicircular ROI of at least 0.2 cm in the longest dimension within the anterior to septal wall. The signal intensity for the blood pool was defined as the mean signal from a circular ROI of at least 0.2 cm drawn in the blood pool (excluding the papillary muscles) of the same mid-ventricular slice. Signal-to-noise ratio of the myocardium (M) and blood (B) was calculated as: SNR_M (B)_ = SI_M (B)_/standard deviation of noise. The contrast-to-noise ratio of myocardium was calculated as: (SI_B_ - SI_M_)/standard deviation of noise.

### Correlation and confirmation of MR findings

Based on the clinical interpretation of the original MR studies, abnormal clinical findings were correlated with surgical reports (3.0 T: *n* = 17; 1.5 T: *n* = 13). Where surgical reports were not available, cardiac catheterization data (3.0 T: *n* = 11; 1.5 T: *n* = 7) or echocardiographic data (3.0 T: *n* = 0; 1.5 T: *n* = 8) were used. Eleven patients had both correlative surgical reports and catheterization data available for review.

### Statistical analysis

Statistical analysis was performed using SAS 9.1 (SAS Inc., Cary Institute, NC) and MedCalc 12.0.1.0 (Mariakerke, Belgium). Discrete values are reported as mean and standard deviation where appropriate. Interobserver agreement was assessed using Cohen’s kappa value. In situations where there is greater prevalence of positive correlation between rater agreement, kappa values may give inconsistent results [20]. Therefore, AC1 statistic was also used in an attempt to address intrinsic limitations of the kappa statistic [21]. A *P*-value <0.05 is considered significant. An AC1 value or kappa value of <0.2, 0.21 to 0.40, 0.41 to 0.60, 0.61 to 0.80, and 0.80 to 1.00 indicates poor, fair, moderate, good and very good agreement, respectively. Continuous variables were tested for normality using the D’Agostino-Pearson test and compared using the Student’s *t*-test. Nonparametric variables were compared using the Mann–Whitney *U* test.

## Results

### Specific Absorption Rate (SAR)

Depending on the imaging technique, SAR values were slightly higher at 3.0 T (Table 2). HR-CEMRA and SSFP cine imaging at 3.0 T generated higher SAR values, but the differences were not statistically different (*P* = 0.20 HR-CEMRA; *P* = 0.52 SSFP cine). Furthermore, all of these values are well below the 4 W/kg limit set by the Federal Drug Administration.

### SSFP cine

Signal-to-noise ratio and blood-myocardial contrast-to-noise ratio were significantly higher at 3.0 T than at 1.5 T. On SSFP cine images, a 2.4-fold increase in signal-to-noise ratio at 3.0 T (45.0 ± 22.3 at 3.0 T vs. 19.0 ± 6.3 at 1.5 T; *P* < 0.001) and a 3.3-fold increase in the contrast-to-noise ratio at 3.0 T (25.7 ± 19.6 at 3.0 T vs. 7.8 ± 5.2 at 1.5 T; *P* < 0.001) were measured.

**Fig. 1:**
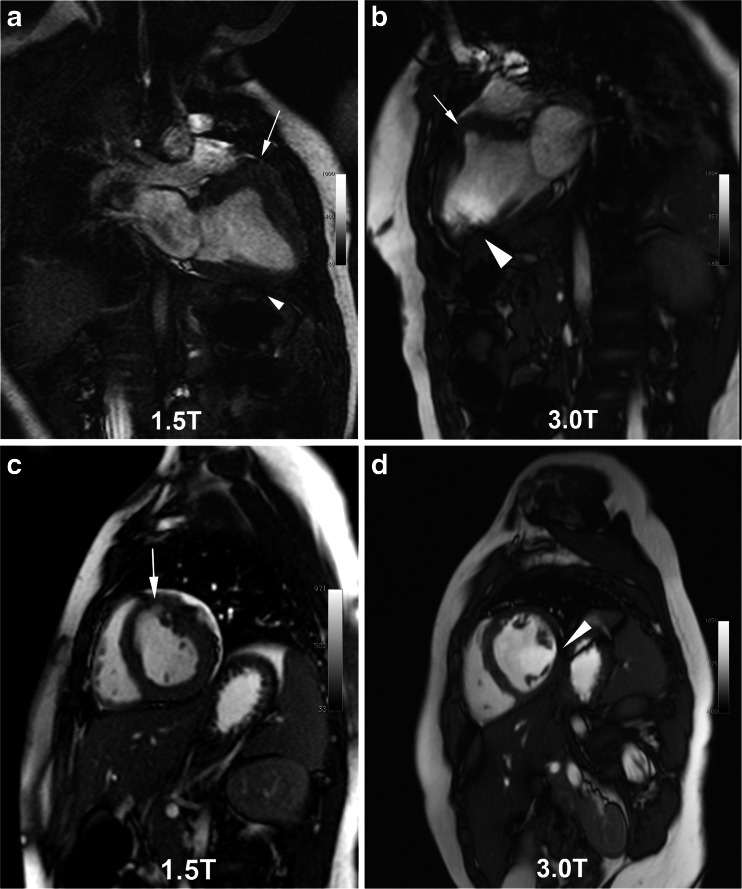
SSFP cine frames at 1.5 T (**a**, **c**) and 3.0 T (**b**, **d**) in the same boy with a low-grade, isointense intramyocardial tumor (*white arrows*), manifest as an aneurysm of the basal anteroseptal wall. Images were obtained three years apart at ages 1.5 years (6.8 kg, 1.5 T) and 4.9 years (16.3 kg, 3.0 T). Image quality was scored as fair at 1.5 T with mild off-resonance banding artifact (*white arrowhead*, **a**). At 3.0 T, the off-resonance artifact is more severe (*white arrowhead*, **b**, **d**) but did not render the images nondiagnostic

**Fig. 2:**
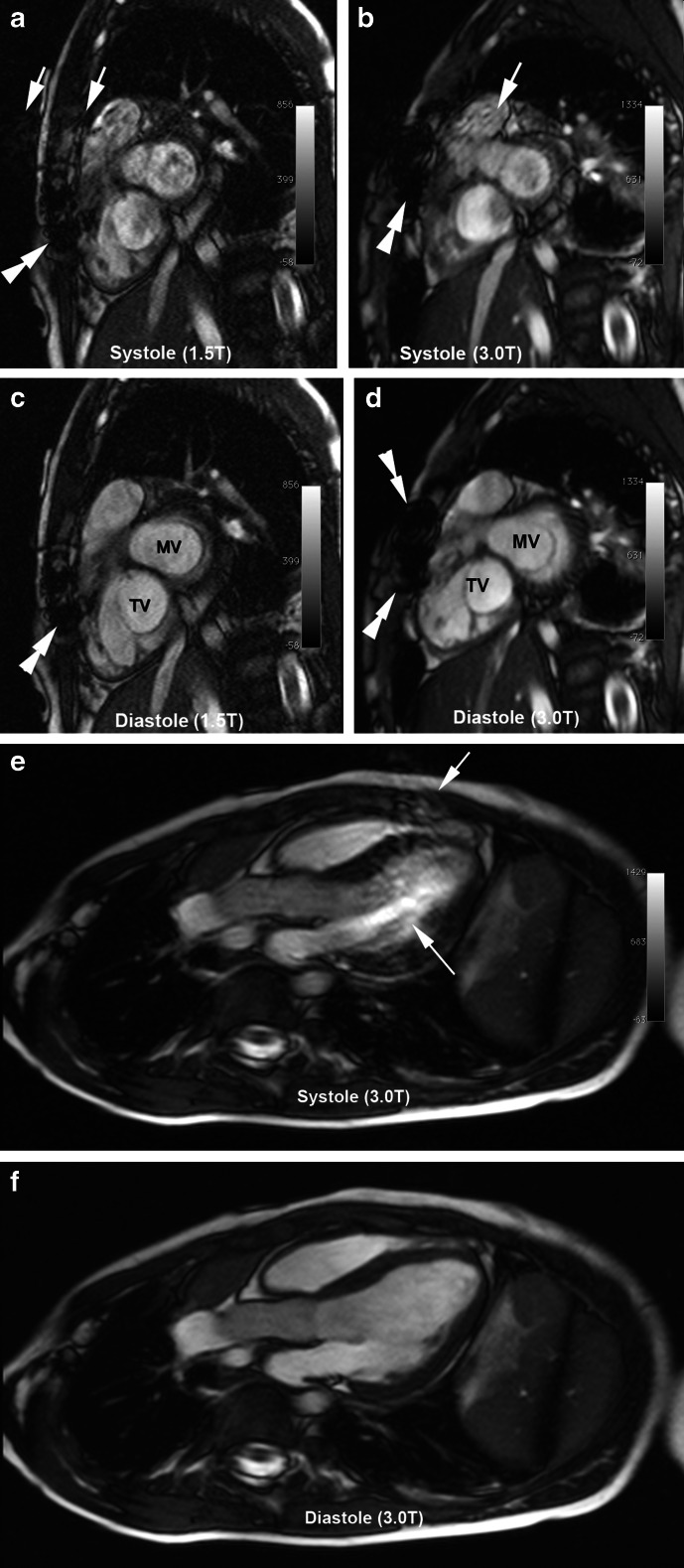
Cardiac phase-specific artifact on SSFP cine at 1.5 T and 3.0 T from a 6-year-old girl with repaired tetralogy of Fallot and whose images were acquired one year apart. Systolic (**a**-**b**) and diastolic (**c**-**d**) basal short axis SSFP at 1.5 T (18 kg, 6.5 years of age) and 3.0 T (16 kg, five years of age) show off-resonance artifacts (*white arrows*) only in the systolic phase at both field strengths. Metal artifacts in the sternum (*double white arrowheads*) are more severe at 3.0 T (**b**, **d**) than at 1.5 T (**a**, **c**). 3.0-T images in systole (**e**) and diastole (**f**) from a 5-year-old boy (23.5 kg) with a basilar tip cerebral aneurysm and aortic coarctation, showing moderate off-resonance artifacts (*white arrows*) related to pulsatile flow during systole only. Image quality in diastole is excellent. *MV* mitral valve, *TV* tricuspid valve

**Fig. 3:**
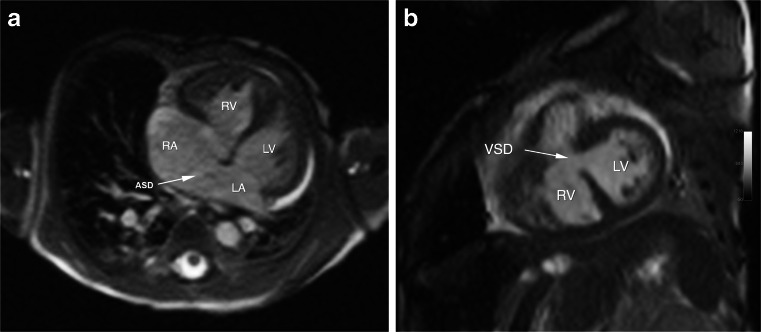
SSFP images at 3.0 T of an 18-day-old (1.9 kg) preterm girl with intracardiac total anomalous pulmonary venous connection (return), large perimembranous ventricular septal defect (VSD), atrial septal defect (ASD), aortic coarctation with arch hypoplasia and large patent ductus arteriosus. SSFP cine images (**a**-**b**) show good intracardiac detail with mild artifacts. There is a 9-mm ASD (**a**, *white arrow*) and a 3.5-mm VSD (**b**, *white arrow*). *LA* left atrium, *LV* left ventricle, *RA* right atrium, *RV* right ventricle

**Fig. 4:**
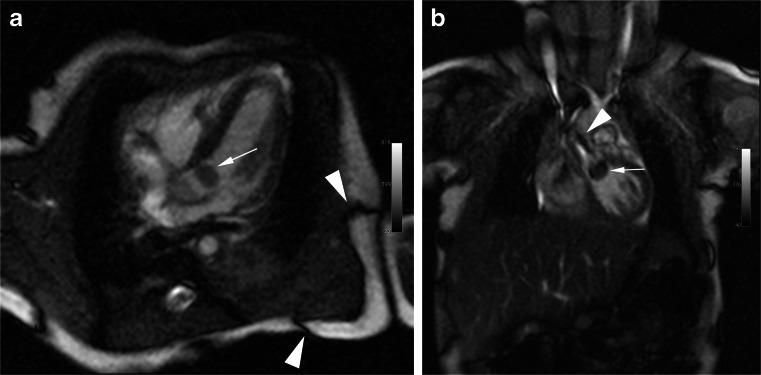
SSFP cine at 3.0 T in a 14-day-old (1.6 kg) boy with biopsy-proven mobile rhabdomyoma (*white arrow *in **a**,**b**) within the left ventricular outflow tract. The center frequency offset in all images was 200 Hz. Dark band artifacts (**a**) and off-resonance artifacts (**b**) from flow in the ascending aorta are indicated by arrowheads. Images were scored as good image quality with mild artifacts

Overall, the average scores for the SSFP cine image quality at 1.5 T and 3.0 T were similar (Table 3), but off-resonance artifact (Fig. 1) was more prevalent at 3.0 T and varied in severity over the cardiac cycle (Fig. 2). Figures 1, 2, 3, 4 provide examples of SSFP cine images at 3.0 T and 1.5 T with representative image quality and artifacts over a range of patient sizes and pathology. Of note, poorer cardiac definition was observed in the basal slices of the SAX SSFP cine images at 3.0 T compared to 1.5 T (Fig. 2). Off-resonance artifact was more prevalent at 3.0 T than at 1.5 T and in 9 patients examined at 3.0 T, the resonance frequency for the SSFP cine images was offset by 50–200 Hz in order to mitigate artifact severity. Frequency-offset maneuvers were felt necessary in one patient at 1.5 T.

**Table 3:** Image quality of SSFP cine imaging and HR-CEMRA of thoracoabdominal vessels

	1.5 T (*n* = 28 patients)	3.0 T (*n* = 28 patients)
SSFP		
Long axis (LAX)	3.2 ± 0.8	3.3 ± 0.6
Short axis (SAX)	3.2 ± 0.7	3.1 ± 0.6
Basal	3.1 ± 0.7	2.8 ± 0.6
Mid	3.3 ± 0.7	3.1 ± 0.7
Apex	3.3 ± 0.7	3.3 ± 0.5
HR-CEMRA		
Overall MRA	3.7 ± 0.5	3.8 ± 0.4
Vessel segments		
Ascending aorta	3.6 ± 0.6	3.7 ± 0.5
Aortic arch	3.8 ± 0.4	3.9 ± 0.3
Descending aorta	3.9 ± 0.3	4.0 ± 0.2
Subclavian	3.7 ± 0.5	3.9 ± 0.3
Carotid	3.8 ± 0.4	3.8 ± 0.4
Pulmonary trunk	3.4 ± 0.7	3.5 ± 0.7
Main PA	3.6 ± 0.6	3.6 ± 0.6
PA branches	3.6 ± 0.5	3.7 ± 0.5
Main PV	3.6 ± 0.6	3.8 ± 0.4
PV branches	3.4 ± 0.7	3.6 ± 0.6
Abdominal aorta	3.9 ± 0.3	3.9 ± 0.3
Celiac trunk	3.8 ± 0.5	3.8 ± 0.4
SMA	3.8 ± 0.5	3.8 ± 0.4
Renal arteries	3.5 ± 0.8	3.5 ± 0.7
Iliac arteries	3.6 ± 0.8	3.6 ± 0.8

*Image quality scores are reported as mean ± SD. *n* = 108 SSFP cine series (*n* = 38 LAX; *n* = 70 SAX) from 28 patients at 1.5 T and *n* = 86 SSFP cine series (*n* = 34 LAX; *n* = 52 SAX) from 28 patients at 3.0 T were analyzed. *n* = 371 vessel segments from 25 exams at 1.5 T and *n* = 414 vessel segments imaged from 28 exams at 3.0 T were analyzed

†Overall image quality for *SSFP cine images* is scored as 1 = poor (nondiagnostic with poorly defined borders and ill-defined intracardiac detail); 2 = fair (diagnostic quality with mild degradation and blurring of cardiac borders); 3 = good (clearly defined cardiac borders with good contrast); 4 = excellent (well-defined cardiac borders with high contrast). Thoracoabdominal vessels *HR-CEMRA* are graded as: 1 = poor--segment could not be confidently evaluated; 2 = fair--segment could be evaluated for structural pathology with moderate confidence; 3 = good--segment could be evaluated for structural pathology with high confidence; 4 = excellent--segment has sharp vessel borders and fine detail can be confidently evaluated. *PV* pulmonary veins (venous), *PA* pulmonary artery, *SMA* superior mesenteric artery

Despite frequency tuning, off-resonance artifact was more prevalent in the basal short axis slices at 3.0 T than at 1.5 T, and when present, these degraded the overall image quality scores. Furthermore, the artifact appeared to be exacerbated by pulsatile flow and was present in some SAX slices only on high flow phases (Fig. 2). When compared to mid-LV and apical SSFP cine images, a higher percentage of the basal SAX SSFP cine images at 3.0 T had degradation and blurring of cardiac borders, resulting in lower image quality scores (Table 3). Additionally, occasional artifacts from sternal wires obscured the RV free wall (Fig. 2) but did not affect the overall interpretation of the image. Although our study did not have sufficient statistical power to detect a difference in overall artifact severity based on quartiles of patient weight, we did observe a general trend toward one grade higher in artifact severity for basal SAX images at 3.0 T in children within the upper weight quartile. When off-resonance artifact was absent, image quality on SSFP cine at 3.0 T was highly diagnostic (Figs. 3 and 4).

### HR-CEMRA

On HR-CEMRA, a 1.8-fold increase in signal-to-noise ratio at 3.0 T (31.7 ± 10.9 at 3.0 T vs. 18.0 ± 9.2 at 1.5 T, *P* = 0.001) and a 1.4-fold increase in contrast-to-noise ratio at 3.0 T (25.2 ± 10.4 at 3.0 T vs. 18.4 ± 9.8 at 1.5 T, *P* = 0.001) were observed.

The highest spatial resolution was achieved in HR-CEMRA at 3.0 T (Table 2). The smallest voxel volumes in HR-CEMRA were 0.2 mm^3^ at 3.0 T and 0.5 mm^3^ at 1.5 T. On average, acquisition times for SSFP cine and HR-CEMRA imaging were comparable at both field strengths (Table 2) and the higher spatial resolution at 3.0 T was achieved via higher parallel acceleration factors and smaller field of view in the frequency encoding direction, relative to 1.5 T.

**Fig. 5:**
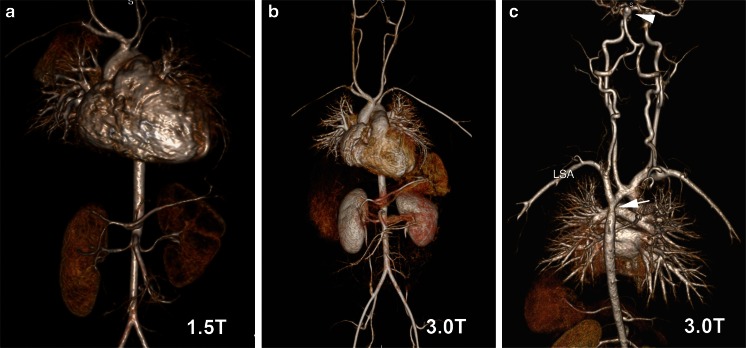
HR-CEMRA with volume-rendered reconstruction at 1.5 T (**a**) and 3.0 T (**b**) in the same 18-month-old (6.8 kg) boy with intramyocardial tumor as shown in Fig. 1. HR-CEMRA with volume-rendered reconstruction (**c**) at 3.0 T in the same 5-year-old boy (23.5 kg) whose SSFP cine is shown in Fig. 2. Note the basilar tip cerebral aneurysm (*white arrowhead*) and aortic coarctation (*white arrow*) distal to the left subclavian artery (LSA)

**Fig. 6:**
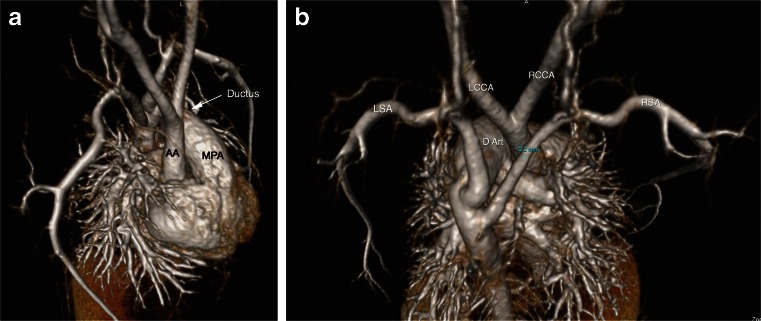
HR-CEMRA at 3.0 T in a 4-day-old (2.7 kg) girl with a type B interrupted aortic arch. Shown are volume-rendered reconstructions from a right superior oblique perspective (**a**) and from a posterosuperior perspective (**b**). The large patent ductus arteriosus (*arrow* in **a**, *D Art* in **b**) is continuous from the main pulmonary artery (MPA) to the distal aortic arch where the left subclavian artery arises. The right subclavian artery is anomalous, originating distal to the left subclavian artery. *AA* ascending aorta, *LCCA* left common carotid artery, *RCCA* right common carotid artery, *LSA* left subclavian artery, *RSA* right subclavian artery

**Fig. 7:**
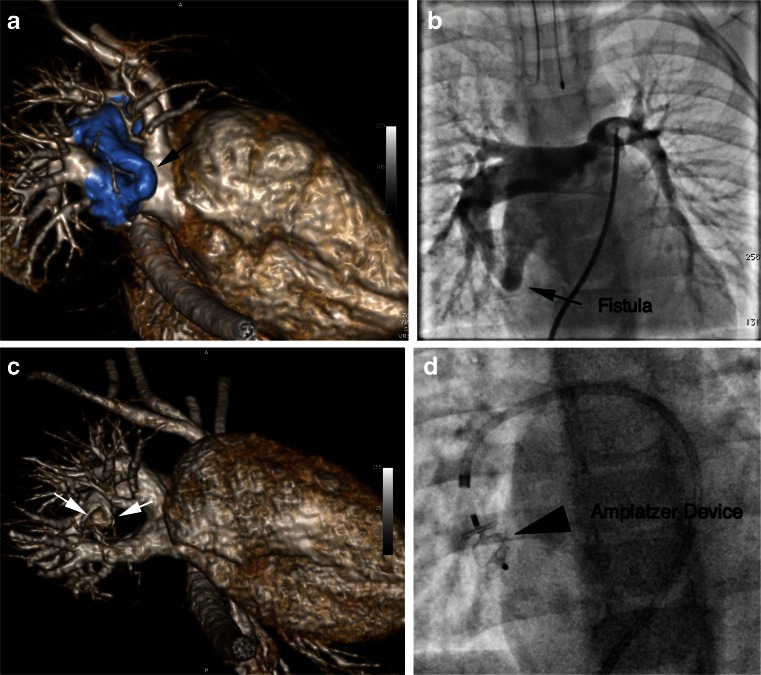
HR-CEMRA at 3.0 T in a 20-month-old (13.3 kg) boy with a right lower lobe pulmonary arteriovenous fistula. Shown are volume-rendered reconstructions from the right inferolateral perspective before (**a**) and following (**c**) occlusion of the fistula by an Amplatzer device. The fistula is clearly shown (highlighted in blue, referenced by *black arrow*) between the right lower lobe pulmonary artery and the right inferior pulmonary vein. In (**c**), the feeding artery is occluded (*white arrows*) by the Amplatzer device and the right inferior pulmonary vein is smaller. Fluoroscopic pulmonary angiography prior to and following device deployment confirmed the presence of the fistula (**b**) and its successful occlusion (**d**)

On HR-CEMRA, the overall image quality scores and percent of images that were rated as good or excellent were similar at both field strengths (Table 3, Fig. 5). Despite the smaller size of vessels in the neonatal cohort, the increased available signal-to-noise ratio at 3.0 T supported the use of high-resolution matrices and image quality scores remained very high, even as patient sizes decreased (Fig. 6). In one patient, the initial diagnosis of pulmonary arteriovenous fistula was made by CEMRA at 3.0 T (Fig. 7) and a follow-up study at 3.0 T post treatment confirmed occlusion of the fistula (Fig. 7).

The average overall artifact scores for HR-CEMRA were similar at 3.0 T and at 1.5 T (Table 4). Artifacts resulting from poor bolus timing were absent. Although uncommon, the most prevalent artifacts at both 3.0 T and 1.5 T resulted from physiologic motion due to incomplete apnea (Table 4). The Mann–Whitney *U* test showed no statistical difference in artifact severity for MRA images obtained at 3.0 T vs. 1.5 T (*P* = 0.72).

**Table 4:** Severity and types of artifacts on SSFP cine and HR-CEMRA images

	1.5 T (*n* = 28 patients)	3.0 T (*n* = 28 patients)
Severity of artifacts		
SSFP		
Long axis (LAX)	1.2 ± 0.5	1.4 ± 0.6
Short axis (SAX)	0.9 ± 0.7	0.9 ± 0.8
Basal	1.2 ± 0.5	1.4 ± 0.7
Mid	1.0 ± 0.7	1.0 ± 0.6
Apex	0.6 ± 0.8	0.3 ± 0.6
HR-CEMRA	1.8 ± 1.6	0.8 ± 0.8
Types of artifacts		
SSFP		
Pulsatile flow	39% (42 of 108 series)	12% (10 of 86 series)
Metal artifacts	19% (20 of 108 series)	6% (5 of 86 series)
Off-resonance (banding)	24% (26 of 108 series)	44% (38 of 86 series)
HR-CEMRA		
Physiologic motion	76% (19 of 25 exams)	68% (19 of 28 exams)
Metal artifacts	8% (2 of 25 exams)	14% (4 of 28 exams)
Parallel imaging	4% (1 of 25 exams)	36% (10 of 28 exams)
Poor bolus timing	0%	0%

**Image quality scores are reported as mean ± SD. *n* = 108 SSFP cine series (*n* = 38 LAX; *n* = 70 SAX) from 28 patients at 1.5 T and *n* = 86 SSFP cine series (*n* = 34 LAX; *n* = 52 SAX) from 28 patients at 3.0 T were analyzed. *n* = 371 vessel segments from 25 exams at 1.5 T and *n* = 414 vessel segments imaged from 28 exams at 3.0 T were analyzed

†Artifacts are graded as 0 = no artifacts; 1 = mild artifacts, not interfering with diagnostic content; 2 = moderate artifacts, degrading diagnostic content; or 3 = severe artifacts, resulting in nondiagnostic images

### Correlation and confirmation of MR findings

Correlation of major abnormal findings with surgical exploration (*n* = 17) or catheterization (*n* = 11) (or both) was excellent. There were no significant false-positive or false-negative findings in any patient with surgical or catheter angiographic correlation. However, correlation of abnormal extracardiac detail with echocardiographic data was poor (*n* = 6) due to less than comprehensive visualization of vessels by ultrasound.

## Discussion

Our results affirm that both high-resolution CEMRA and diagnostic SSFP cardiac cine images were obtained reliably at 3.0 T in neonates and small children (ages 3 days to 8 years, weighing 1.4–25.0 kg). With patient-specific frequency tuning [22], off-resonance SSFP artifacts were manageable and the cine images were generally diagnostic. Further, signal-to-noise ratio and contrast-to-noise ratio are significantly increased at 3.0 T relative to 1.5 T and CEMRA with very high-resolution matrices was routinely achievable.

The clinical value of MR is well established for a variety of cardiovascular disorders [2, 23–26]. Whereas in adults, the manifestations of cardiac disease can generally be defined by focusing on the heart, children with CHD typically have both intracardiac and extracardiac anomalies. Therefore, the ability to visualize extracardiac vascular anomalies in addition to intracardiac morphological abnormalities is crucial. Although echocardiography is readily available, it may leave several morphological and hemodynamic questions unresolved. Cardiac CT with either single- or dual-source technology can yield high spatial resolution images with increasingly small radiation doses. However, sub-milliSievert doses are achievable only in the most ideal situations (low body mass index, sinus rhythm with excellent beta blockade). Exposure to radiation, however low the dose, should be considered only when alternative methods are not available. Furthermore, neonates and infants may have heart rates that challenge the temporal resolution of even dual-source CT scanners. With MRI, the temporal resolution of cine imaging can be adjusted to cope with even the fastest heart rates [27]. MRI has been shown to be a versatile, noninvasive and non-radiating technique for the evaluation of neonates, infants and children, including those who are critically ill [2, 28, 29].

In young children, the small caliber of the heart and blood vessels requires higher spatial resolution than in adults [23, 25] for comparable detail. In Cartesian MRI, spatial resolution is expressed in the three logical dimensions (frequency encoding, in plane-phase encoding and through-plane phase encoding) that define the volume element, or voxel. In 3-D imaging, all three orthogonal directions are equally relevant in determining voxel size. Each voxel dimension may be expressed as the ratio of the field of view (FOV) to the matrix size. For CEMRA of the thorax in adults at 1.5 T, voxel volumes on the order of 2–2.5 mm^3^ are not atypical even for acquisitions regarded as high resolution [30, 31]. For CEMRA of the thorax and abdomen in children at 1.5 T, voxel volumes of 2.7 mm^3^ have been described [32] and in neonates, to our knowledge, the smallest voxels previously described have been 1.6 mm^3^ [33]. Whereas for adults and larger children, spatial resolution as previously reported may be entirely adequate, for small children with diminutive central vessels, the requirements for high spatial resolution are much more stringent. Earlier reports of CEMRA in the abdomen with limited spatial resolution in children found disappointing results relative to CT [34]. We therefore sought to maximize spatial resolution in small children by performing CEMRA at 3.0 T with fine matrices and aggressive parallel acquisition factors. Our highest spatial resolution in the smallest children at 3.0 T is 8 times greater than previously reported in the literature and 2.5 times greater than what we achieved at 1.5 T. As spatial resolution is increased, signal-to-noise ratio decreases and this generally limits the available spatial resolution at 1.5 T. We were able to realize unprecedented spatial resolution for CEMRA in small children because the higher available signal-to-noise ratio at 3.0 T can be used to maintain adequate signal-to-noise ratio even as the voxel size is diminished.

For SSFP cine imaging, we aimed for less aggressive increments in spatial resolution relative to 1.5 T, because increasing spatial resolution generally involves the use of a longer time repetition (TR) and a corresponding increased sensitivity to off resonance banding artifact. We used the maximum allowable flip angle in all cases to achieve the shortest TR possible. Because imaging of very small children requires high spatial resolution and small FOV, the short TR in SSFP imaging is often in conflict, which may be a limiting factor for SSFP imaging at 3.0 T. Despite these challenges, the smallest achievable SSFP voxel volume in our study was 2.4 mm^3^ at 3.0 T and 2.8 mm^3^ at 1.5 T.

At 3.0 T, the boost in baseline signal-to-noise ratio can be exploited to support higher resolution [17, 18]. Our signal-to-noise ratio measurements showed several interesting features. Theoretically, signal-to-noise ratio increases linearly with field strength. In practice, signal-to-noise ratio is affected by voxel size, receiver bandwidth, RF coil sensitivity profile, and sequential vs. parallel image acquisition [5, 6]. Despite these trade-offs, we observed an increase in myocardial and blood signal-to-noise ratio by a factor of 2.4 and an increase in contrast-to-noise ratio by a factor of 3.3. Although consistent with published literature on SSFP cine imaging at 3.0 T in the adult population [35–37], it should be noted that our signal-to-noise ratio/contrast-to-noise ratio measurements were made at end-diastole, where off-resonance is typically not prevalent at either field strength. With this caveat in mind, the higher prevalence of off-resonance artifact at the base of the heart may well tip this balance more in favor of 1.5 T. In our study, the acquisition matrix for SSFP cine at 3.0 T was slightly coarser than that typically used at 1.5 T in order to minimize the TR per line and mitigate off-resonance effects. Despite the higher prevalence of artifacts on images acquired at 3.0 T, the image quality was comparable between 3.0 T and 1.5 T as reflected by well-defined borders and high myocardium to blood pool contrast ratio on 3.0-T images.

Another factor that may contribute to the high measured signal-to-noise ratio at 3.0 T relates to the frequency response function of the SSFP signal [38]. As the frequency of the spins drifts away from resonance, the signal amplitude initially increases before decreasing dramatically at +/− (1/2TR) to form a dark band. The signal from blood in the neighborhood of this off-resonance condition, therefore, may actually increase and in doing so may yield a higher value for signal-to-noise ratio and blood-myocardial contrast-to-noise ratio. However, this is not a desirable condition because it represents an unstable state from which any increase in resonance offset will result in dark band artifact. Price et al. [22] recently addressed the importance of stabilizing the frequency for cardiac SSFP imaging in neonates at 3.0 T. Although the authors used a different scanner platform, they noted a significant drift in the resonant frequency in normal, spontaneously breathing neonates at 3.0 T and emphasized the importance of local shimming and adjustment of resonance frequency offsets. In our study population, dark band artifacts occurred more frequently in the LAX and basal SAX views. We did not observe a particular relationship between subject size and the presence of dark band artifacts.

Conventional wisdom suggests that SSFP imaging is better at 1.5 T than at 3.0 T. This is routinely the case. However, in our experience, local frequency tuning provided diagnostic SSFP MR imaging at 3.0 T in a wide range of patient size, age and complex cardiac as well as vascular abnormalities with some increase (additional 2–3 min) in overall exam time. At 3.0 T, frequency tuning was performed about 30% of the time; whereas, at 1.5 T, only 4% of the studies required frequency tuning. At 3.0 T (as compared to 1.5 T), the higher signal-to-noise ratio as manifested by well-defined myocardial borders and contrast is impacted negatively by the increased presence of artifacts.

Several limitations in our study merit discussion. We performed a retrospective analysis with a cross-sectional study design. Ideally, our patients would undergo the same sequence protocol at 1.5 T so that intra-individual comparisons can be performed. However, ethical considerations relating to the study of infants and neonates under anesthesia preclude non-indicated MR scans to be performed purely for head-to-head comparative research purposes. Further, due to the unique nature of the underlying spectrum of pathologies, each exam was tailored to the specific indication and therefore corresponding views and imaging sequences were not always available for comparison in all patients. Importantly, our criteria for assigning patients either to 3.0 T or 1.5 T results in clear selection bias. We assigned very small children to 3.0 T, if available, to achieve the highest resolution CEMRA. In doing so, we imposed asymmetry in the 3.0-T and 1.5-T patient groups, such that the 1.5-T group is on average older and larger. However, there is substantial overlap in the older and larger patients of both 1.5-T and 3.0-T cohorts, making head-to-head comparisons reasonable. Additionally, we recognize that with parallel imaging [17, 18], measurement of signal-to-noise ratio becomes complicated. However, the design of our study made it difficult to implement the signal-to-noise ratio measurement methods described by Kellman et al. [39] and Reeder et al. [40]. Although the method used in our paper to estimate signal-to-noise ratio is less than ideal, others have compared our practical alternative approach to other methods available and have found it to produce reasonable estimates [41, 42].

## Conclusion

Diagnostic MR imaging and MR angiography at 3.0 T was reliably achieved in children with CHD over a broad range of ages (3 days to 8 years) and sizes (1.4–25.0 kg). Artifacts on SSFP cine were cardiac phase-specific and more prevalent at 3.0 T, but the highest-resolution CEMRA protocols were implemented successfully in the smallest patients at 3.0 T. In neonates where very high spatial resolution CEMRA was of primary clinical relevance, 3.0 T was highly reliable and should be considered in this patient population where available.
